# Understanding the effects of neighborhood disadvantage on youth psychopathology

**DOI:** 10.1017/S0033291721005080

**Published:** 2023-05

**Authors:** Sarah L. Carroll, Kelly L. Klump, S. Alexandra Burt

**Affiliations:** Department of Psychology, Michigan State University, East Lansing, MI, USA

**Keywords:** Disadvantage, genotype-environment interplay, psychopathology

## Abstract

**Background:**

In 1942, Shaw and McKay reported that disadvantaged neighborhoods predict youth psychopathology (Shaw & McKay, 1942). In the decades since, dozens of papers have confirmed and extended these early results, convincingly demonstrating that disadvantaged neighborhood contexts predict elevated rates of both internalizing and externalizing disorders across childhood and adolescence. It is unclear, however, *how* neighborhood disadvantage increases psychopathology.

**Methods:**

Our study sought to fill this gap in the literature by examining the Area Deprivation Index (ADI), a composite measure of Census tract disadvantage, as an etiologic moderator of several common forms of psychopathology in two samples of school-aged twins from the Michigan State University Twin Registry (*N* = 4815 and 1030 twin pairs, respectively), the latter of which was enriched for neighborhood disadvantage.

**Results:**

Across both samples, genetic influences on attention-deficit hyperactivity problems were accentuated in the presence of marked disadvantage, while nonshared environmental contributions to callous-unemotional traits increased with increasing disadvantage. However, neighborhood disadvantage had little moderating effect on the etiology of depression, anxiety, or somatic symptoms.

**Conclusions:**

Such findings suggest that, although neighborhood disadvantage does appear to serve as a general etiologic moderator of many (but not all) forms of psychopathology, this etiologic moderation is phenotype-specific.

Neighborhood disadvantage (e.g. Census-tract poverty, crime) has been linked to numerous forms of youth psychopathology, with children from impoverished communities demonstrating higher rates of nearly every mental health disorder compared to their peers in wealthier neighborhoods. Indeed, youth in disadvantaged neighborhoods experience elevated rates of depression, anxiety, somatic symptoms, and attention-deficit hyperactivity disorder (ADHD) (e.g. Xue, Leventhal, Brooks-Gunn, and Earls, 2005), are more likely to be deemed aggressive in peer reports (Kupersmidt, Griesler, DeRosier, Patterson, & Davis, 1995), and to exhibit low levels of prosocial behavior (Lichter, Shanahan, & Gardner, 2002). These findings persist throughout childhood and adolescence and are observed across racial/ethnic identities (Gorman–Smith & Tolan, 1998).

While disadvantage in the broader neighborhood is related to lower family socioeconomic status (e.g. household income, parental education), they are not synonymous. Indeed, typical associations among neighborhood and familial disadvantage are relatively small (*r*s 0.2–0.4; Mode, Evans, and Zonderman, 2016). Moreover, their consequences may increment one another. For example, neighborhood poverty has been found to predict child antisocial behavior even after controlling for familial poverty (Kupersmidt et al., 1995). Similarly, neighborhood residential stability continues to predict adolescent internalizing and externalizing outcomes when controlling for both familial occupational status and parental lifetime diagnoses (Buu et al., 2009). In the same study, changes in neighborhood residential stability from early childhood through adolescence also predicted adolescent mental health (Buu et al., 2009). Such results point to a unique contribution of neighborhood characteristics, both concurrently and longitudinally, to young people's mental health outcomes, above and beyond the effects of socioeconomic deprivation and other stressors occurring at the family level. These findings are consistent with Bronfenbrenner's ecological systems theory, which views individual development as being embedded in multiple contexts. Each of these contexts (e.g. family, school, neighborhood) is believed to contribute uniquely to youth development (Bronfenbrenner, 1988), meaning that the effects of disadvantage on youth psychopathology cannot be fully understood without considering indices beyond those existing at the family level.

Despite these robust associations and theoretical considerations, only a few studies have examined the mechanisms linking neighborhood disadvantage to youth psychopathology. These biometric GxE studies leverage the differing degrees of genetic similarity between identical and fraternal twins to determine the genetic and environmental contributions to the trait under study, as well as whether these contributions shift based on a moderating variable. Results from these studies suggest that neighborhood disadvantage exerts its effects ‘under the skin’ by altering the respective genetic and environmental variances for psychopathology. This phenomenon has been observed most consistently for youth antisocial behavior. Several studies conducted in different labs using different samples (Burt, Klump, Gorman-Smith, & Neiderhiser, 2016; Cleveland, 2003; Tuvblad, Grann, & Lichtenstein, 2006) have reported that neighborhood disadvantage serves as an etiologic moderator of antisocial behavior, with shared environmental influences (experiences common to children in the same family; e.g. similar parenting) accounting for considerably more variance in impoverished neighborhoods relative to wealthy neighborhoods. These results are robust to the conceptualization of neighborhood (e.g. Census tract, 1 km, 5 km) and to informant-reports of neighborhood problems (i.e. administrative Census data, maternal and neighbor informant-reports) (Burt, Pearson, Carroll, Klump, & Neiderhiser, 2019b). Such findings are typically interpreted as evidence of a bioecological genotype-environment interaction (GxE), which predicts that environmental influences will predominate in disadvantaged contexts whereas genetic influences will predominate in ‘average, expectable’ environments (Bronfenbrenner & Ceci, 1994).

It remains unclear, however, whether this pattern of moderation extends to other forms of youth psychopathology. Extant biometric GxE studies of disadvantage have largely restricted their analyses to antisocial behavior to the exclusion of other forms of child psychopathology (e.g. depression, anxiety, ADHD), or they have examined *family-level* indices, rather than neighborhood-level indices, as moderators (e.g. Middeldorp et al., 2014). Although one such study found an increase in genetic contributions to depression with greater neighborhood disadvantage, participants were adults (Strachan, Duncan, Horn, & Turkheimer, 2017). We thus do not know whether the pattern of bioecological GxE by neighborhood disadvantage observed consistently for child antisocial behavior extends to other forms of psychopathology. It seems *a priori* likely that it would, given the high levels of comorbidity among youth antisocial behavior, ADHD, and internalizing problems, as well as the overlapping etiologies of these disorders (Cosgrove et al., 2011; Tackett, Waldman, Van Hulle, & Lahey, 2011; Thapar, Harrington, & McGuffin, 2001).

However, neighborhood disadvantage may not act as an environmental moderator for all forms of psychopathology, or it may alter etiology in a different way. The diathesis-stress GxE model hypothesizes that environmental stressors will activate genetic vulnerabilities to psychiatric symptoms, with genetic influences predominating in disadvantaged environments (Ingram & Luxton, 2005). This pattern of moderation stands in direct contrast to the predictions of the bioecological GxE model (environmental influences increase and genetic influences decrease with disadvantage). In short, because no prior study of GxE has examined the mechanisms linking neighborhood disadvantage to youth psychopathology broadly, it is unclear both whether and how neighborhood disadvantage might affect the etiologies of disorders beyond antisocial behavior. Given the prevalence and public health burden of such disorders (Costello, Mustillo, Erkanli, Keeler, & Angold, 2003), particularly in impoverished communities (Leventhal & Brooks-Gunn, 2000), this represents an important gap in the literature.

The goal of the present study was to fill this gap by examining neighborhood disadvantage as an etiologic moderator of multiple forms of youth psychopathology in two independent twin samples, one of which was enriched for neighborhood disadvantage. We examined neighborhood disadvantage as an etiologic moderator of three broad forms of child psychopathology (ADHD, anxiety, depression). We also examined callous-unemotional (CU) traits, a newly added specifier for diagnoses of Conduct Disorder, as etiologic moderation by neighborhood has not yet been evaluated for CU traits despite their documented links to conduct problems. Based on prior research pointing to consistent etiologic moderation by neighborhood disadvantage, we hypothesized that disadvantage would moderate the etiology of each outcome. We did not have specific hypotheses as to which model of GxE might be most important for internalizing symptoms or ADHD, although we hypothesized that CU traits would be subject to bioecological moderation given their link to antisocial behavior.

## Methods

### Participants

The current study used two samples within the population-based Michigan State University Twin Registry (MSUTR; Burt and Klump, 2013; Klump and Burt, 2006): the Twin Study of Behavioral and Emotional Development in Children (TBED-C) and the Michigan Twins Project (MTP). In both studies, parents provided informed consent for themselves and their children. The TBED-C includes both a population-based subsample (*n* = 528 families) and an independent ‘at-risk’ subsample enriched for neighborhood disadvantage (*n* = 502 families). Additional inclusion criteria for the ‘at-risk’ subsample required that participating twin families lived in modestly to severely disadvantaged Census tracts. Mean household income was $ 76 329 (s.d. = $ 45 650) in the population-based sample and $ 55 652 (s.d. = $ 31 088) in the at-risk sample. Other recruitment details are included in prior publications (e.g. Burt, Clark, Pearson, Klump, and Neiderhiser, 2019a). Across the TBED-C, the twins ranged from 6 to 11 years old (mean = 8.06, s.d. = 1.45) and were 49% female. Families identified as White: 81%, Black: 10%, Latino(a): 1%, Asian: 1%, Indigenous: 1%, multiracial: 6%. There were 224 monozygotic (MZ) male twin pairs, 211 dizygotic (DZ) male pairs, 202 MZ female pairs, 206 DZ female pairs, and 187 DZ opposite-sex pairs.

The primary aim of the on-going, population-based MTP is to collect health data on Michigan-born twins (current *N* ~ 12 000 twin pairs) for either data analysis or to select families for follow-up research (Burt & Klump, 2012). Because TBED-C families were recruited out of the MTP, they were excluded from the MTP sample for these analyses. To maximize comparability to the TBED-C, however, we restricted inclusion to MTP twin pairs in middle childhood. The final sample for this study consisted of 4815 twin pairs (mean age = 8.79, s.d. = 2.38, range 5–12 years; 49.1% female; mean household income = $ 90 252, s.d. = $ 57 573). Families identified as White: 81%, Black: 8%, Latino(a): 2%, Asian: 1%, Indigenous: 0.5%, multiracial: 4.5%. There were 649 MZ male twin pairs, 852 DZ male pairs, 622 MZ female pairs, 802 DZ female pairs, and 1875 DZ opposite-sex pairs.

### Neighborhood disadvantage

Neighborhood disadvantage was assessed via the Area Deprivation Index (ADI), a composite measure of 17 indices of community disadvantage (e.g. Census-tract poverty rate, income disparity; see online Supplementary Table S1 for a list of all measures included and Singh (2003) for additional details). We recreated Kind and Buckingham's index of disadvantage, assessed via Census data collected from 2008 to 2012 (Nearly all participants completed the assessment of psychopathology within this time span) (Kind & Buckingham, 2018). The measures were weighted according to the factor loadings identified by Singh (2003), and weighted variables were summed to create a deprivation index score for each Census tract. Families were assigned a percentile indicating the level of deprivation in their Census tract relative to that of all Census tracts in Michigan. The mean ADI was 37.84 (s.d. = 27.00) in the MTP and 42.47 (s.d. = 26.18) in the TBED-C and ranged from 1 to 100 in both samples. As the ADI is a community-based measure derived from Census tract data, there is no shared method variance between disadvantage and mothers' reports of child psychopathology (detailed below).

### Child psychopathology

Mothers in the MTP completed the Strengths and Difficulties Questionnaire (SDQ; Goodman, 2001), a 25-item measure in which parents rate the extent to which a series of statements describe the child's behavior over the past 6 months using a three-point scale (0 = not true to 2 = certainly true). We examined the Hyperactivity/Inattention (e.g. easily distracted; four items; *α* = 0.84), Emotional Symptoms (e.g. nervous; five items; *α* = 0.64), and Prosocial Behavior (e.g. kind, helpful; five items; *α* = 0.77) scales. The prosocial scale was reverse-scored, with higher scores indicating fewer prosocial behaviors. Data were available for 94% of the twins. Psychometric studies have found the SDQ to have satisfactory test-retest reliability (*r* ⩾ 0.75 for the subscales used here) and to be highly correlated with other parent-report measures, including the Child Behavior Checklist (CBCL) (e.g. Muris, Meesters, and van den Berg, 2003).

In the TBED-C, we obtained both mother and teacher reports of twin behavioral and emotional problems. The twins' mothers completed the CBCL and the twins' teacher(s) the corresponding Achenbach Teacher Report Form (TRF) (Achenbach & Rescorla, 2001). We included the Attention Problems, Anxious/Depressed, Withdrawn/Depressed, and Somatic Complaints subscales from the TRF and CBCL (all *α* ⩾ 0.7). On these scales, informants rated the extent to which a series of statements described the child's behavior over the past 6 months using a three-point scale (0 = never to 2 = often/mostly true). Mothers also completed the Callous, Uncaring, and Unemotional subscales from the Inventory of Callous-Unemotional Traits (ICU; Kimonis et al., 2008; all *α* ⩾ 0.7). On the ICU, mothers used a four-point scale to rate how well a series of statements described the child's current behavior (0 = definitely false to 3 = definitely true).

Maternal informant-reports from the CBCL and ICU were available for 99% and 58% of the twins, respectively (the ICU was added to the protocol roughly 2.5 years into the study). The teachers of 119 twins were not available for assessment, and our final teacher participation rate across the TBED-C was 83%. Completed TRFs were available for 75% of the twins. Mother and teacher reports were averaged to form multi-informant composites of child psychopathology, consistent with prior research (e.g. Burt *et al*. 2019a, 2019b). When combined, CBCL/TRF data were available for 2057 participants (99.9%). The study protocol was designed to minimize rater-contrast effects as much as possible, given that mothers and most teachers (84%) reported on both twins. For example, mothers first completed all questionnaires on Twin 1, then engaged in a series of other activities, and subsequently completed all questionnaires on Twin 2. Put differently, informants did not report on both twins' characteristics back-to-back.

### Data analyses

Classical twin studies leverage the difference in the proportion of segregating genes shared between monozygotic or MZ twins (who share 100% of their genes) and dizygotic or DZ twins (who share an average of 50% of their segregating genes) to estimate the relative contributions of genetic and environmental influences to the variance within observed behaviors (phenotypes). Phenotypic variance is decomposed into three of four components: additive genetic (A), dominant genetic (D; nonadditive or gene-to-gene interactive effects, which yield MZ twin correlations more than twice those of DZ twins), shared environmental (C), and nonshared environmental (E), the latter of which indexes environmental exposures that serve to differentiate twins raised in the same family (e.g. peer groups). Because A, C, and D are estimated using the same information (i.e. differences in sibling similarity by genetic relatedness), it is only possible to simultaneously estimate two of the three in traditional twin designs. As a result, either C or D was fixed to zero in all univariate twin analyses. More information on twin studies is provided elsewhere (Neale & Cardon, 1992).

For this study, we fitted the ‘univariate GxE’ model (Purcell, 2002), as shown in Supplementary Fig. S1, the most appropriate GxE model when the twins are perfectly concordant on the moderator (Van der Sluis, Posthuma, & Dolan, 2012). In this model, the variance decompositions of each disorder were modeled as a function of neighborhood disadvantage. Disadvantage was first entered in a means model of the outcome, controlling for the main effect of ADI on each form of psychopathology. Moderation was then modeled on the residual variance (i.e. variance in a given form of psychopathology that does not overlap with neighborhood disadvantage). The least restrictive of these models allows for linear moderation of A, C or D, and E contributions. We then fitted the more restrictive no moderator model along with relevant sub-models depending on the results of the full moderation model, constraining the linear moderators to be zero and evaluating the reduction in model fit. Next, we conducted a series of supplemental GxE analyses using the Nuclear Twin Family Model, which incorporates data from the parents in addition to the twins and thus allows us to directly model passive gene-environment correlation (rGE). Because twins were concordant on the moderator, we were unable to fit a model examining GxE in the presence of rGE (Van Hulle & Rathouz, 2015). Fortunately, the young age of the twins precludes the possibility of neighborhood niche-picking (as children do not choose where they live), meaning we would not expect there to be active or evocative rGE effects that could bias the results of our GxE analyses.

M*plus* 8.0 (Muthén & Muthén, 1998-2019) was used to fit the GxE models to the data using Full-Information Maximum-Likelihood techniques. When models are fit to raw data, means, variances, and covariances are first freely estimated to obtain a baseline fit index (minus twice the log-likelihood; −2lnL). Fit was evaluated using the Akaike's information criterion (AIC; Akaike, 1987), the Bayesian information criterion (BIC; Raftery, 1995), and the sample-size adjusted Bayesian information criterion (SABIC; Sclove, 1987). The lowest AIC, BIC, and SABIC among a series of nested models is considered best, and the best-fitting model was indicated by the lowest AIC, BIC, and SABIC values, as well as non-significant change in chi-square, for at least 3 of the 4 fit indices. In all analyses, ADI was examined as a continuous moderator ranging from 0 to 1. Sex and age were regressed out of all twin data, consistent with prior recommendations (McGue & Bouchard, 1984). Finally, as it is recommended that unstandardized or absolute parameter estimates be presented in moderation models (Purcell, 2002), the log-transformed and residualized psychopathology scores were standardized to have a mean of zero and a standard deviation of one prior to analysis to facilitate interpretation of the unstandardized value.

## Results

Descriptive statistics are presented in online Supplementary Table S2 and described in online Supplementary Results. Also shown in the Supplement are the twin intraclass correlations (see online Supplementary Results and Table 1), which provided preliminary evidence that the pattern of moderation varied across phenotypes. We confirmed this impression through formal tests of etiologic moderation, evaluating ADI as a continuous moderator.

**Table 1.** Twin intraclass correlations at lower and higher neighborhood disadvantage

Sample	Phenotype	Advantaged				Disadvantaged			
		*r*MZ (95% CI)	*N*	*r*DZ (95% CI)	*N*	*r*MZ (95% CI)	*N*	*r*DZ (95% CI)	*N*
MTP	Hyperactivity/ Inattention	0.52 (0.44–0.59)	382	0.04 (−0.02 to 0.10)	1170	0.64 (0.58–0.69)	458	0.12 (0.06–0.18)	1035
TBED-C	Attention Problems	0.48 (0.37–0.57)	225	0.31 (0.20–0.42)	259	0.64 (0.55–0.72)	178	0.20 (0.09–0.31)	307
MTP	Emotional Symptoms	0.53 (0.45–0.60)	381	0.21 (0.16–0.26)	1164	0.60 (0.54–0.66)	446	0.31 (0.25–0.36)	1034
TBED-C	Anxious/ Depressed	0.39 (0.27–0.50)	225	0.38 (0.27–0.48)	259	0.38 (0.25–0.50)	178	0.38 (0.28–0.47)	307
TBED-C	Withdrawn/ Depressed	0.48 (0.37–0.57)	225	0.21 (0.09–0.32)	259	0.43 (0.30–0.54)	178	0.24 (0.13–0.34)	307
TBED-C	Somatic Symptoms	0.60 (0.51–0.68)	225	0.37 (0.26–0.47)	259	0.49 (0.37–0.59)	178	0.34 (0.24–0.44)	307
MTP	Prosocial Behavior	0.63 (0.57–0.69)	389	0.31 (0.26–0.36)	1175	0.53 (0.46–0.59)	453	0.31 (0.25–0.36)	1033
TBED-C	Callous	0.55 (0.39,.68)	95	0.14 (−0.03 to 0.30)	131	0.41 (0.25–0.55)	124	0.29 (0.17–0.41)	221
TBED-C	Uncaring	0.49 (0.32–0.63)	96	0.13 (−0.04 to 0.29)	131	0.53 (0.39–0.64)	127	0.08 (−0.05 to 0.21)	222
TBED-C	Unemotional	0.34 (0.15–0.51)	96	0.01 (−0.16 to 0.18)	131	0.29 (0.12–0.44)	126	0.01 (−0.12 to 0.14)	221

**Fig. 1.**
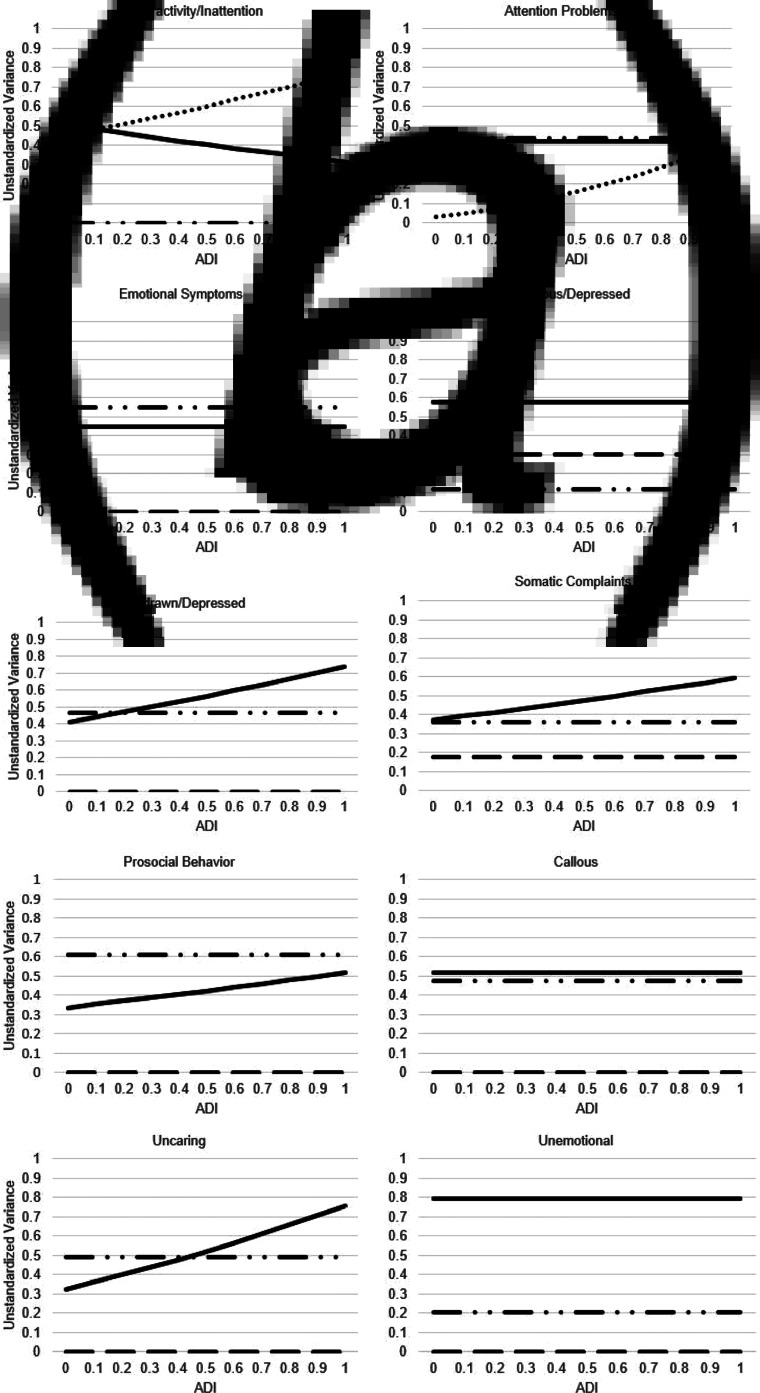
Etiologic moderation of (**a**) ADHD, (**b**) Emotional Problems, and (**c**) Callous-Unemotional Traits by neighborhood disadvantage. The latter is indexed by the Area Deprivation Index (ADI), a composite measure of disadvantage at the Census tract level. 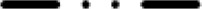, Additive genetic effects; 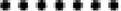, Nonadditive genetic effects; 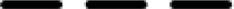, Shared environmental effects; 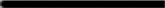, Nonshared environmental effects.

Table 2 contains model fit statistics. Table 3 contains the parameter estimates for the full and best-fitting linear moderation models. Given the documented presence of nonadditive genetic influences on ADHD (Burt, 2009), as well as the ICCs indicating the same pattern in these data (see Table 1), we fitted an ADE model to our measures of ADHD. The DE moderation model fit the data best in the MTP (Fig. 1a), with D increasing significantly and E decreasing somewhat with greater disadvantage. In the TBED-C, we similarly found evidence of D moderation (the D moderation only model best fit the data), although the E moderator could be constrained to zero.

**Table 2.** Biometric GxE fit indices

Phenotype	−2lnL	χ*^2^* (*df*)	AIC	BIC	SABIC
MTP Hyperactivity/Inattention					
Linear ADE moderation	17097.86	–	17113.87	17162.32	17136.90
**Linear DE moderation**	**17097.86**	**0.00 (1)**	**17111.87**	**17154.26**	**17132.02**
Linear D moderation	17104.76	6.90† (2)	17116.76	17153.10	17134.03
No moderation	17109.02	11.16† (3)	17119.01	17149.29	17133.41
TBED-C Attention Problems					
Linear ADE moderation	5302.74	–	5318.74	5357.77	5332.36
Linear AD moderation	5302.80	0.06 (1)	5316.80	5350.95	5328.72
**Linear D moderation**	**5304.86**	**2.12 (2)**	**5316.87**	**5346.14**	**5327.09**
No moderation	5311.28	8.54† (3)	5321.28	5345.67	5329.79
MTP Emotional Symptoms					
Linear ACE moderation	16955.76	–	16971.76	17020.20	16994.78
Linear C moderation	16959.06	3.30 (2)	16971.06	17007.39	16988.33
**No moderation**	**16962.76**	**7.00 (3)**	**16972.75**	**17003.03**	**16987.14**
TBED-C Anxious/Depressed					
Linear ACE moderation	5345.08	–	5361.09	5400.12	5374.71
**No moderation**	**5346.12**	**1.04 (3)**	**5356.11**	**5380.51**	**5364.63**
TBED-C Withdrawn/Depressed					
Linear ACE moderation	5370.42	–	5386.41	5425.45	5400.04
Linear CE moderation	5370.42	0.00 (1)	5384.42	5418.58	5396.34
Linear C moderation	5379.60	9.18† (2)	5391.61	5420.88	5401.83
**Linear E moderation**	**5371.90**	**1.48 (2)**	**5383.90**	**5413.18**	**5394.12**
No moderation	5384.20	13.78† (3)	5394.20	5418.59	5402.71
TBED-C Somatic Complaints					
Linear ACE moderation	5269.88	–	5285.88	5324.92	5299.51
**Linear E moderation**	**5271.28**	**1.40 (2)**	**5283.29**	**5312.56**	**5293.51**
No moderation	5277.56	7.68 (3)	5287.56	5311.96	5296.08
MTP Prosocial Behavior					
Linear ACE moderation	16999.90	–	17015.90	17064.36	17038.94
Linear C moderation	17010.80	10.90† (2)	17022.79	17059.14	17040.07
**Linear E moderation**	**17006.04**	**6.14**† **(2)**	**17018.04**	**17054.39**	**17035.32**
Linear CE moderation	17002.20	2.30 (1)	17016.20	17058.60	17036.36
No moderation	17017.68	17.78† (3)	17027.67	17057.96	17042.07
TBED-C Callous					
Linear ACE moderation	3185.66	–	3201.66	3236.57	3211.18
Linear C moderation	3188.20	2.54 (2)	3200.21	3226.40	3207.35
Linear E moderation	3185.80	0.14 (2)	3197.81	3224.00	3204.95
**No moderation**	**3189.44**	**3.78 (3)**	**3199.44**	**3221.26**	**3205.39**
TBED-C Uncaring					
Linear ACE moderation	3202.40	–	3218.41	3253.32	3227.93
**Linear E moderation**	**3202.40**	**0 (2)**	**3214.41**	**3240.60**	**3221.55**
No moderation	3211.30	8.90† (3)	3221.29	3243.12	3227.24
TBED-C Unemotional					
Linear ACE moderation	3254.98	–	3270.98	3305.89	3280.49
**No moderation**	**3255.36**	**0.38 (3)**	**3265.37**	**3287.18**	**3271.31**

†Significant change in χ^2^ at *p* < 0.05. Bolded text indicates which model best fits the data, based on all fit indices provided.

**Table 3.** Unstandardized path and moderation parameter estimates for the full linear moderation model and best-fitting moderation model. 95% confidence intervals are reported below each estimate

Phenotype	Paths			Linear Moderators		
	*a*	*d*	*e*	A1	D1	E1
MTP Hyperactivity/Inattention						
Linear ADE moderation	0.00 (0.00–0.00)	0.67* (0.60–0.73)	0.71* (0.64–0.77)	0.00 (0.00–0.00)	0.21* (0.07–0.35)	−0.15~ (−0.30 to 0.01)
**Linear DE moderation**	0.00 (0.00–0.00)	0.67* (0.60–0.73)	0.71* (0.64–0.77)	**–**	0.21* (0.07–0.35)	−0.15~ (−0.30 to 0.01)
No moderation	0.00 (0.00–0.00)	0.75* (0.73 to 0.77)	−0.65* (−0.67 to −0.62)	**–**	**–**	**–**
TBED-C Attention Problems						
Linear ADE moderation	0.72* (0.58–0.86)	0.19 (−0.03 to 0.41)	0.64* (0.54–0.73)	−0.37 (−0.88 to 0.14)	0.68* (0.49–0.87)	0.02 (−0.21 to 0.25)
Linear AD moderation	0.72* (0.59–0.84)	0.19 (−0.01 to 0.39)	0.64* (0.59–0.70)	−0.37 (−0.88 to 0.14)	0.69* (0.49–0.89)	**–**
**Linear D moderation**	0.66* (0.51–0.81)	0.18 (−0.22 to 0.58)	0.65* (0.59–0.70)	**–**	0.44* (0.12–0.76)	**–**
No moderation	0.60* (0.39–0.82)	0.47* (0.03–0.90)	0.65* (0.59–0.71)	**–**	**–**	**–**
	*a*	*c*	*e*	A1	C1	E1
MTP Emotional Symptoms						
Linear ACE moderation	0.70* (0.62–0.79)	−0.06~ (−0.12 to 0.00)	0.70* (0.63–0.77)	0.03 (−0.15 to 0.22)	0.46* (0.12–0.80)	−0.09 (−0.19 to 0.02)
**No moderation**	0.74* (0.71–0.77)	0.00 (0.00–0.00)	−0.66* (−0.70 to −0.63)	**–**	**–**	**–**
TBED-C Anxious/Depressed						
Linear ACE moderation	0.17 (−0.35 to 0.68)	0.60* (0.37–0.84)	0.78* (0.69–0.87)	0.43 (−0.36 to 1.21)	−0.14 (−0.69 to 0.42)	−0.06 (−0.25 to 0.12)
**No moderation**	0.35 (−0.03 to 0.72)	0.55* (0.41–0.69)	0.76* (0.70–0.81)	**–**	**–**	**–**
TBED-C Withdrawn/Depressed						
Linear ACE moderation	0.66* (0.38–0.94)	−0.24 (−0.65 to 0.17)	0.65* (0.55–0.75)	−0.02 (−0.69 to 0.65)	0.68* (0.00–1.36)	0.21~ (−0.03 to 0.44)
**Linear E moderation**	0.68* (0.55–0.81)	0.00 (−0.31 to 0.31)	0.64* (0.57–0.72)	**–**	**–**	0.22* (0.08–0.35)
No moderation	0.69* (0.55–0.82)	0.00 (−0.32 to 0.32)	0.73* (0.67–0.79)	**–**	**–**	**–**
TBED-C Somatic Complaints						
Linear ACE moderation	0.71* (0.49–0.93)	0.33 (−0.10 to 0.76)	0.59* (0.49–0.68)	−0.28 (−0.78 to 0.21)	0.19 (−0.49 to 0.88)	0.22* (0.03–0.40)
**Linear E moderation**	0.61* (0.40–0.82)	0.41* (0.14–0.68)	0.61* (0.51–0.70)	**–**	**–**	0.16* (0.01–0.31)
No moderation	−0.61* (−0.83 to −0.39)	0.41* (0.12–0.70)	0.68* (0.61–0.74)	**–**	**–**	**–**
MTP Prosocial Behavior						
Linear ACE moderation	0.81* (0.75–0.87)	−0.19* (−0.33,-0.04)	0.56* (0.50–0.63)	−0.18 (−0.45 to 0.09)	0.70* (0.36,1.04)	0.19* (0.04–0.35)
**Linear E moderation**	0.78* (0.70–0.86)	0.00 (−0.25 to 0.25)	0.58* (0.51–0.64)	**–**	**–**	0.14* (0.05–0.23)
No moderation	0.77* (0.68–0.87)	0.00 (−0.32 to 0.32)	0.63* (0.57–0.69)	**–**	**–**	**–**
TBED-C Callous						
Linear ACE moderation	0.71* (0.47–0.96)	−0.02 (−0.32 to 0.28)	0.62* (0.47–0.77)	−0.09 (−0.61 to 0.43)	0.34 (−0.27 to 0.96)	0.22 (−0.10 to 0.54)
**No moderation**	0.70* (0.54–0.86)	0.00 (−0.36 to 0.36)	0.72* (0.63–0.80)	**–**	**–**	**–**
TBED-C Uncaring						
Linear ACE moderation	0.70* (0.52–0.88)	0.00 (0.00–0.00)	0.58* (0.44–0.72)	0.00 (−0.35 to 0.34)	0.00 (0.00–0.00)	0.29* (0.03–0.56)
**Linear E moderation**	0.70* (0.62–0.77)	0.00 (0.00–0.00)	0.58* (0.44–0.72)	**–**	**–**	0.29* (0.00–0.59)
No moderation	0.71* (0.63–0.79)	0.00 (0.00–0.00)	0.71* (0.63–0.79)	**–**		**–**
TBED-C Unemotional						
Linear ACE moderation	0.47* (0.07–0.87)	0.00 (−0.14 to 0.14)	0.85* (0.69–1.02)	−0.01 (−0.69 to 0.68)	0.00 (−0.45 to 0.45)	0.06 (−0.19 to 0.31)
**No moderation**	0.47* (0.37–0.56)	0.00 (0.00–0.00)	0.88* (0.83–0.94)	**–**	**–**	**–**

Bold font and asterisk, *p* < 0.05. ~, *p* < 0.10.

Because prior research has identified significant shared environmental contributions to all other common forms of youth psychopathology (e.g. Burt, 2009), we fitted an ACE model to our other measures. For internalizing, the no moderation model provided the best fit to the MTP data. All moderator values could be constrained to zero, and the plotted lines were thus flat (see Fig. 1b). This finding persisted to the Anxious/Depressed scale in the TBED-C, in that the no moderation model best fit the data. However, we did observe a small moderating effect on both Withdrawn/Depressed and Somatic Complaints, with nonshared environmental contributions increasing with greater disadvantage.

For reverse-scored Prosocial Behavior in the MTP sample, we found that nonshared environmental influences increased slightly with increasing disadvantage (Fig. 1c). Additive genetic influences were large and shared environmental influences were small regardless of the level of disadvantage, and both the A and C moderators could be constrained to zero. These results were partially replicated in the TBED-C. Disadvantage moderated the nonshared environmental contribution to scores on the Uncaring (*p* < 0.05) scale, with little-to-no evidence of E moderation for Unemotional or Callous scores.

## Discussion

Our goal was to evaluate neighborhood disadvantage as an etiologic moderator of multiple forms of youth psychopathology in two independent samples, one of which was enriched for disadvantage. Results indicated that although disadvantage does appear to serve as a general etiologic moderator of many (but not all) forms of psychopathology, the specific type of moderation varied across disorders. For ADHD, disadvantage exerted a consistent diathesis-stress effect, augmenting the nonadditive genetic variance in both samples. By contrast, neighborhood disadvantage did not accentuate genetic contributions to internalizing. For internalizing symptoms, the A, C, and E moderators could all be constrained to zero in the MTP, suggesting little-to-no etiologic moderation. Analyses in the TBED-C similarly indicated little moderation of internalizing symptoms, although there was evidence that nonshared environmental contributions to Withdrawn/Depressed and Somatic Complaints increased slightly with increasing disadvantage. Although this finding persisted to two scales within the TBED-C, the fact that it was specific to only one sample indicates that, at most, there is limited evidence of GxE by disadvantage for internalizing. Regardless, these findings are not consistent with the diathesis-stress GxE effects observed for ADHD. Finally, we observed a small increase in nonshared environmental variance with increasing disadvantage for CU traits and related low prosociality, although unlike with internalizing symptoms, this effect was observed across both samples. However, this effect was restricted to Uncaring symptoms of CU traits in the TBED-C, the scale most highly correlated with reverse-scored Prosocial Behavior. To our knowledge, this is the first study to examine the moderating effects of neighborhood disadvantage on children's internalizing or on CU traits at any age.

Despite our use of two independent twin samples, there are several limitations to this study. First, both samples were restricted to twins in middle childhood. This consideration is relevant given meta-analytic work indicating that the genetic and environmental contributions to many forms of psychopathology shift across early development (Burt, 2009). Further research is needed to clarify whether these findings, particularly for internalizing, persist to other age groups. While internalizing disorders, particularly anxiety, do emerge during childhood for some youth (Cartwright-Hatton, McNicol, & Doubleday, 2006), their prevalence increases dramatically during adolescence (e.g. Davis, Votruba-Drzal, and Silk, 2015). Future studies should thus re-examine these GxE for internalizing in adolescent samples. We also note that the no moderation model best fit the Emotional Symptoms data in the MTP, despite the presence of significant C moderation (*p* < 0.05) in the full ACE model. Future research is needed to clarify whether internalizing disorders are in fact subject to a bioecological moderating effect, given the inconsistent results across samples.

Next, we relied on composite reports of child psychopathology provided by the twins' mothers and teachers. While the use of composites provides a fuller conceptualization of youth outcomes across contexts (Achenbach, McConaughy, & Howell, 1987), it may obscure differences by informant. However, supplemental analyses of mother reports of child psychopathology yielded the same pattern of GxE as observed for mother-teacher composites, with little evidence of moderation for any form of internalizing and significant D moderation for Attention Problems (moderator = 0.44, *p* < 0.05). Results are reported in online Supplementary Tables S3 and S4. For teacher reports, there was also little evidence of moderation for Anxious/Depressed or Withdrawn/Depressed. Teacher-reported scores on Somatic Complaints remained highly skewed (skew statistic > 2), even after applying log and cube-root transformations. Because prior simulations (Purcell, 2002) demonstrate that skewed outcome measures can yield spurious GxE findings (both false positives and false negatives), we were unable to obtain interpretable results for this subscale. For teacher-reported Attention Problems, we observed a moderate-to-large increase in D with greater disadvantage in both the DE moderation and D only moderation models (moderators were 0.60 and 0.59, respectively, both *p* < 0.05). In short, the pattern of moderation was consistent across conceptualizations of youth psychopathology.

Next, estimates of A and D contributions to ADHD were somewhat inconsistent across samples. We compared the respective fits of the univariate ACE, ADE, and AE models and found that the ADE model best fit the data in the MTP, while the AE model fit best in the TBED-C (see online Supplementary Table S5). The point estimate for D in the TBED-C, however, was moderate in magnitude and significantly different than zero (0.44, *p* < 0.05). This is likely related to power, as prior analyses have found these parameters difficult to detect even in large samples (Rietveld, Posthuma, Dolan, & Boomsma, 2003). As a robustness check, we re-ran the moderation analyses using the AE model and found that, in both samples, A increased with greater disadvantage (see online Supplementary Tables S6–S7). Thus, genetic influences were consistently amplified as disadvantage increased. Future studies in larger samples are needed to definitively conclude which type of genetic influence is subject to moderation.

Next, our study focused on disadvantage at the Census-tract level. Given that disadvantage comes in myriad forms (e.g. neighborhood, familial) that are not highly correlated (Mode et al., 2016), future studies should examine whether the pattern of etiologic moderation identified here persists to other aspects of disadvantage. In addition, while we examined multiple dimensions of internalizing pathology (e.g. anxiety, depression), the SDQ and CBCL each included a single scale for ADHD that measured symptoms of inattention and hyperactivity/impulsivity together. While prior work has found moderate-to-high correlations between these symptom dimensions during middle childhood (Greven, Asherson, Rijsdijk, & Plomin, 2011; McLoughlin, Rijsdijk, Asherson, & Kuntsi, 2011), and considerable overlap in their etiologies (McLoughlin et al., 2011), future research is needed to confirm that our findings for ADHD persist to each symptom dimension individually.

Finally, one assumption of our model is that *r*MZ is no more than twice as large as *r*DZ in magnitude (Purcell, 2002). For ADHD, we addressed this assumption by fitting an ADE moderation model, given the consistent evidence of D in ADHD. Internalizing psychopathology, by contrast, is not generally influenced by D (Burt, 2009). To evaluate the extent to which relevant assumptions in the univariate ACE moderation model were violated in these data, we incorporated parental self-report data to fit a nuclear twin family model (NTFM), which allows us to simultaneously estimate A, D, and ‘C’ (termed S or F in the NTFM). Additional details are available in Supplementary Materials. Results did not suggest nonadditive genetic contributions to Anxious/Depressed or Somatic Complaints, suggesting that the ACE model was appropriate in those cases. We did observe significant D contributions to Withdrawn/Depressed (see online Supplementary Table S8). However, subsequent NTFM moderation analyses replicated the increase in E with greater disadvantage (see online Supplementary Table S9). Additional NTFM analyses yielded moderation results for Anxious/Depressed, Somatic Complaints, and Attention Problems that were consistent with those observed in the Purcell models (also shown in online Supplementary Table S9), indicating that unmeasured passive rGE are unlikely to be driving our results. Thus, limitations of the ACE moderation model do not appear to compromise the validity of our findings for internalizing or ADHD. Because the ICU was added into the protocol 2.5 years after the study began, we did not have sufficient parent data to run NTFM analyses for CU traits. However, additional GxE analyses using an ADE moderation model yielded the same pattern of moderation for all three scales as was observed in the ACE moderation models (i.e., increase in E with greater disadvantage for Uncaring, little-to-no moderation of Callous or Unemotional; see online Supplementary Tables S10 and S11), meaning our results are robust to model type.

Despite these limitations, our study sheds light on the impact of broader neighborhood disadvantage, or disadvantage existing beyond the family context, on the genetic and environmental underpinnings of youth mental health. Our results indicate that neighborhood disadvantage is a relatively consistent etiologic moderator of multiple forms of psychopathology in middle childhood. When combined with prior results for antisocial behavior in these and other data (e.g. Burt et al., 2019a, 2019b), our results also clearly indicate that the moderating effects of neighborhood disadvantage are phenotype-specific. We have now observed, across multiple samples, evidence of bioecological moderation for youth antisocial behavior, diathesis-stress moderation for ADHD, and non-shared environmental moderation for CU traits, but little-to-no moderation of internalizing symptoms.

Such findings have numerous implications. First, they could suggest that externalizing symptoms may be subject to more robust etiologic moderation by neighborhood disadvantage during middle childhood relative to internalizing symptoms. Should this be true, we may expect etiologic and even phenotypic patterns of comorbidity between internalizing and externalizing to also differ across contexts. Further research is needed to determine the ways in which children's mental health problems manifest differentially across contexts. Alternately, these results could imply that the moderating effects of disadvantage differ by disorder because the mechanism of effect is different. This is entirely possible, given that the ADI is a composite of 17 disadvantage indicators. Toxicant exposure, for example, could operate ‘under the skin’, activating genetic predispositions for ADHD, while child-specific exposure to community violence could amplify the importance of siblings' nonshared experiences in their development of prosocial tendencies. As our results indicated a more robust contribution of nonadditive genetic influences, particularly in the MTP, on ADHD in disadvantaged neighborhoods, perhaps the exposures conducive to the development of inattention/hyperactivity problems differentially impact the expression of genes that operate in a dominant fashion. Regardless, these findings suggest that genetically similar children are particularly likely to be concordant for ADHD diagnoses when living in impoverished neighborhoods.

Overall, our results underscore the profound role that neighborhood disadvantage can play in shaping the origins of children's behavioral outcomes while also indicating that this role varies across different forms of psychopathology. Future studies should seek to clarify the ‘active ingredients’ of disadvantaged neighborhoods that underlie their moderating effects on each disorder under study.

## Supplementary material

For supplementary material accompanying this paper visit https://doi.org/10.1017/S0033291721005080. click here to view supplementary material
