# Supplementary material for: Understanding the effects of neighborhood disadvantage on youth psychopathology
Source: Psychol Med. 2022 Feb 16;53(7):3036–46. doi: 10.1017/S0033291721005080 (PMC9378764; doi:10.1017/S0033291721005080)
Supplement: Supplementary file 1 [file S0033291721005080sup001.docx]

**Supplementary Methods**

To assess whether the assumptions of the univariate ACE model held for internalizing psychopathology in our sample, we incorporated responses to the Achenbach Adult Self-Report (ASR; Achenbach & Rescorla, 2003) from parents in TBED-C on scales that were analogous to those on the CBCL/TRF (i.e., Anxious/Depressed, Withdrawn/Depressed, and Somatic Complaints). By incorporating data on the parents of the twins as well as the twins themselves, the nuclear twin family model provides four pieces of information on which to base parameter estimates: the covariance between MZ twins, the covariance between DZ twins, the covariance between parents, and the covariance between parents and children. This additional information allows us to estimate several parameters on top of those estimated by the classical twin model. First, we can directly estimate assortative mating, or the extent to which biological parents resemble one another, in the model and account for its effects accordingly. Next, we are able to disambiguate two general types of shared environmental influences: 1) those that create similarity between siblings, but not between parents and their children (termed S; e.g., exposure to common peers, school, and experiences of similar parenting across siblings), and 2) those that are passed via vertical “cultural transmission” between parents and their offspring (termed F; e.g., socioeconomic status, social mores). The model then allows us to capitalize on this newfound individuation of the various types of shared environmental influences by directly estimating the covariance between F and genetic influences, or passive rGE effects (Keller, Medland, & Duncan, 2010).

In the NTFM, we are able to estimate at most four of the five relevant etiologic influences (A, E, D, S, and F). We are thus required to fix one of these estimates to zero in each model. For Anxious/Depressed, Withdrawn/Depressed, and Somatic Complaints, we compared the respective fits of 7 models (ASFE, ADFE, ADSE, ASE, AFE, ADE, and AE) to determine which best fit the data for each scale. Fit was assessed using four indices: the AIC, BIC, SABIC, and root mean square error of approximation (RMSEA; Hooper, Coughlan, & Mullen, 2008). The best-fitting model was indicated by the lowest or most negative values for at least three of the four fit indices. As an additional verification, we compared the same series of models for Attention Problems.

Next, we conducted a series of nuclear twin family GxE analyses using the best-fitting model identified for each outcome measure. We dichotomized ADI at the median and fitted a multigroup model that allowed us to obtain parameter estimates for families experiencing lower and higher levels of neighborhood disadvantage, respectively, consistent with prior GxE work using the NTFM (Burt, Klump, Gorman-Smith, & Neiderhiser, 2016). These analyses thus allow us to more definitively evaluate whether the pattern of GxE observed via the Purcell model reflects shifts in the importance of genetic influences and/or environmental exposures rather than shifts in the importance of passive rGE and/or assortative mating.

There are two important assumptions undergirding the NTFM. First, although the model accommodates the possibility of assortative mating, it assumes that assortative mating stems from primary phenotypic assortment, in which mates choose each other based on phenotypic similarity, and does not allow for other forms of assortative mating (e.g., social homogamy, in which mates choose each other due to environmental similarity). Second, A and E are assumed to influence all traits to some extent (Burt et al., 2016).

**Supplementary Results**

Descriptive Statistics

In the MTP, boys scored higher on Hyperactivity/Inattention (Cohen’s *d* effect size was .46, *p*<.001), and lower on Prosocial Behavior (*d* = -.33, *p*<.001) and Emotional Symptoms (*d* = -.14, *p*<.001). In the TBED-C, boys scored higher on Attention Problems, Callous, and Uncaring (*d*s were .50, .20, and .24, respectively, *p*<.05), and modestly higher on Anxious/Depressed and Withdrawn/Depressed (*d*s were .09 and .12, respectively, *p*<.05). Such findings are consistent with prior results indicating that the female preponderance of internalizing symptoms emerges during puberty (Hayward & Sanborn, 2002).

Neighborhood disadvantage was modestly associated with Hyperactivity/Inattention, Emotional Symptoms, and Prosocial Behavior in the MTP, as well as with Attention Problems, Withdrawn/Depressed, Somatic Symptoms, and Callous subscales in the TBED-C (see Table S2). Although these associations are small, we note that the extent to which a moderator is correlated with an outcome does not foreshadow the presence or magnitude of etiologic moderation (Purcell, 2002). Although the twins in the TBED-C were otherwise not included in our MTP analyses, we did examine those participants (N=1,358) to confirm expected associations between the SDQ and the CBCL/TRF and ICU. Hyperactivity was correlated .54 across the SDQ and CBCL/TRF. Anxious/Depressed, Withdrawn/Depressed, and Somatic Symptoms on the CBCL/TRF were correlated .47, .34, and .35, respectively, with Emotional Symptoms on the SDQ. Lastly, Callous, Uncaring, and Unemotional symptoms were correlated .28, .35, and .12, respectively, with the reverse-scored Prosocial Behavior scale. Correlations between mother and teacher reports in the TBED-C for Anxious/Depressed, Withdrawn/Depressed, Somatic Complaints, and Attention Problems were .20, .27, .08, and .44, respectively, *p*<.01. This pattern of correlations is consistent with those of other studies, with externalizing scores somewhat more highly correlated across respondents than internalizing ones (e.g., Grigorenko et al., 2010).

Preliminary evidence of GxE

To preliminarily evaluate the possible presence of etiologic moderation, we dichotomized ADI at the median and compared the twin intraclass correlations at low and high levels of disadvantage (note that the ADI was evaluated continuously in the formal models). Correlations are reported in Table 1. Across both samples, the MZ correlation for ADHD symptoms was larger than the DZ correlation, indicating the likely presence of genetic influences regardless of level of disadvantage. That said, MZ similarity increased with disadvantage, suggesting either a decrease in E and/or an increase in A or D. For internalizing scales, by contrast, there was little preliminary evidence of etiologic moderation. MZ and DZ twin correlations remained largely unchanged across level of disadvantage. Unexpectedly, the correlations for Anxious/Depressed were identical in MZ and DZ twins, consistent with prior studies finding that environmental influences on internalizing symptoms can predominate prior to adolescence (e.g., Rice, Harold, & Thapar, 2002). For prosocial/CU traits, there was somewhat more preliminary evidence of moderation. MZ twin similarity for prosocial behavior in the MTP decreased with increasing disadvantage, while DZ twin similarity remained constant, indicating possible moderation of the shared and/or nonshared environmental contributions. For Callous scores in the TBED-C, MZ twin similarity was significantly greater than DZ twin similarity at low disadvantage, but not high disadvantage, suggesting a possible increase in environmental variance. By contrast, the ICCs for Uncaring and Unemotional symptoms did not appear to shift with increasing disadvantage.

**Table S1:** Measures included in Area Deprivation Index.

| ***Measure*** |  |
| --- | --- |
|  | 1. Percent of population aged 25 and older with <9 years of education |
|  | 1. Percent of population aged 25 and older with at least a high school diploma |
|  | 1. Percent of population aged 16 and older in white-collar occupations |
|  | 1. Median family income |
|  | 1. Income disparity (ratio of households with <$10,000 income to households with ≥$50,000 income) |
|  | 1. Median home value |
|  | 1. Median gross rent |
|  | 1. Median monthly mortgage |
|  | 1. Percent of housing units owned by occupiers |
|  | 1. Percent of population aged 16 and older who are unemployed |
|  | 1. Percent of families below poverty level |
|  | 1. Percent of population below 150% of the poverty threshold |
|  | 1. Percent of households with children under age 18 headed by a single parent |
|  | 1. Percent of households without a motor vehicle |
|  | 1. Percent of households without a telephone |
|  | 1. Percent of occupied housing units without complete plumbing |
|  | 1. Percent of households with more than 1 person per room |

*Note.* Participating families’ ADI scores were determined by the level of deprivation in their Census tract based on all indices listed above. For additional details, see Singh (2003) and Kind & Buckingham (2018).

**Table S2**: Descriptive statistics.

| **Sample** | **Phenotype** | Mean (SD) | Range | Phenotypic *r* with disadvantage (95% CI) |
| --- | --- | --- | --- | --- |
|  |  |  |  |  |
| MTP | Hyperactivity/Inattention | 2.18 (2.29) | 0 - 8 | **.14*** (.12 - .16) |
| TBED-C | Attention Problems | 4.70 (5.19) | 0 - 32 | **.11*** (.07 - .15) |
|  | |  | |  |
| MTP | Emotional Symptoms | 1.81 (1.87) | 0 - 10 | **.11*** (.09 - .14) |
| TBED-C | Anxious/  Depressed | 2.59 (2.61) | 0 – 19.50 | -.03 (-.07 - .02) |
| TBED-C | Withdrawn/  Depressed | 1.15 (1.62) | 0 - 13 | **.07*** (.03 - .11) |
| TBED-C | Somatic Symptoms | .93 (1.30) | 0 - 13 | **.06*** (.02 - .10) |
|  | |  | |  |
| MTP | Prosocial Behaviorǂ | 1.74 (1.92) | 0 - 10 | **.06*** (.04 - .09) |
| TBED-C | Callous | 6.82 (4.65) | 0 - 31 | **.08*** (.02 - .14) |
| TBED-C | Uncaring | 5.32 (3.80) | 0 - 24 | .05 (-.01 - .11) |
| TBED-C | Unemotional | 4.09 (3.16) | 0 - 15 | -.04 (-.10 - .02) |

ǂReverse-scored. Bold font and an asterisk, *p* is significantly different than zero at <.05.

**Table S3:** Biometric GxE fit indices for mother reports of youth psychopathology in TBED-C.

| **Phenotype** | | | | **-2lnL** | | | | | ***χ²* (*df*)** | | | | **AIC** | | | **BIC** | | | **SABIC** | |
| --- | --- | --- | --- | --- | --- | --- | --- | --- | --- | --- | --- | --- | --- | --- | --- | --- | --- | --- | --- | --- |
| Attention Problems |  | | | | | | |  | | | |  | | |  | | | | |  |
| Linear ADE moderation | | | | | | 5326.50 | |  | | | | - | | | 5342.50 | | | 5381.46 | | 5356.06 |
| Linear AD moderation | | |  | | 5326.56 | | | | | | .06 (1) | | | 5340.56 | | | 5374.66 | | | 5352.43 |
| **Linear D moderation** | | |  | | **5327.64** | | | | | **1.14 (2)** | | | | **5339.63** | | | | **5368.86** | | **5349.80** |
| **No moderation** | |  | | | | | **5330.60** | | | **4.10 (3)** | | | | **5340.59** | | | | **5364.95** | | **5349.07** |
| Anxious/Depressed | | | | | | | | | | | | | | | | | | | | |
| Linear ACE moderation | | | | 5304.92 | | | | | - | | | | 5320.93 | | | 5359.90 | | | 5334.49 | |
| **No moderation** | | | | **5305.32** | | | | | **.40 (3)** | | | | **5315.32** | | | **5339.68** | | | **5323.80** | |
| Withdrawn/Depressed | | | | | | | | | | | | | | | | | | | | |
| Linear ACE moderation  Linear CE moderation | | | | 5335.90  5336.82 | | | | | -  .92 (1) | | | | 5351.90  5350.82 | | | 5390.86  5384.92 | | | 5365.46  5362.68 | |
| Linear C moderation | | | | 5339.32 | | | | | 3.42 (2) | | | | 5351.32 | | | 5380.54 | | | 5361.49 | |
| **Linear E moderation**  No moderation | | | | **5338.48**  5343.82 | | | | | **2.58 (2)**  7.92† (3) | | | | **5350.47**  5353.82 | | | **5379.70**  5378.17 | | | **5360.64**  5362.29 | |
| Somatic Complaints | | | | | | | | | | | | | | | | | | | | |
| Linear ACE moderation | | | | 5210.74 | | | | | - | | | | 5226.73 | | | 5265.70 | | | 5240.29 | |
| Linear E moderation  **No moderation** | | | | 5213.00  **5213.20** | | | | | 2.26 (2)  **2.46 (3)** | | | | 5225.00  **5223.20** | | | 5254.23  **5247.56** | | | 5235.17  **5231.68** | |

†Significant change in chi-square at *p*<.05.

**Table S4:** Unstandardized path and moderation parameter estimates for mother reports of youth psychopathology in TBED-C.

| **Phenotype** | **Paths** | | **Linear Moderators** |
| --- | --- | --- | --- |
|  | **a d e** | | **A1 D1 E1** |
| Attention Problems  Linear ADE moderation  Linear AD moderation  **Linear D moderation**  **No moderation** | **.60* .39* .68***  **.59* .39* .68***  **.46* .49* .68***  .43~ **.59* .69*** | | -.39~ **.44*** .02  -.39~ **.46*** -  - .20 -   - - - |
|  | **a c** | **e** | **A1 C1 E1** |
| Anxious/Depressed  Linear ACE moderation  **No moderation**  Withdrawn/Depressed  Linear ACE moderation  **Linear E moderation**  No moderation  Somatic Complaints  Linear ACE moderation  Linear E moderation  **No moderation** | .21 **.56***  .16 **.60***  **.68*** -.02  **.66*** .00  **.66*** .08  **.66* .45***  **.53* .52***  **.52* .52*** | **.78***  **.78***  **.69***  **.69***  **.75***    **.63***  **.66***  **.67*** | -.08 .08 .00  - - -  -.25 **.59*** .16  - - **.15***  - - -  **-.33*** .14 .09  - - .03  - - - |

Bold font and asterisk, *p*<.05. ~, *p*<.10.

**Table S5:** Biometric fit indices for ADHD symptoms from standard univariate models.

| **Sample** | **-2lnL** | | | | ***χ²* (*df*)** | | **AIC** | | | **BIC** | **SABIC** | |
| --- | --- | --- | --- | --- | --- | --- | --- | --- | --- | --- | --- | --- |
| MTP Hyperactivity/Inattention | |  | |  | |  | |  | | | |  |
| ACE model  **ADE model**  AE model | | | 19891.76  **19811.40**  19891.76 |  | | -  -  80.36† (1) | | 19899.75  **19819.40**  19897.75 | 19924.54  **19844.19**  19916.34 | | | 19911.83  **19831.48**  19906.81 |
| TBED-C Attention Problems | | | | | | | | | | | | |
| ACE model  ADE model  **AE model** | 5623.98  5622.40  **5623.98** | | | | -  -  **1.58 (1)** | | 5631.97  5630.39  **5629.97** | | | 5651.72  5650.14  **5644.78** | 5639.01  5637.43  **5635.25** | |

†Significant change in chi-square at *p*<.05.

**Table S6:** Biometric GxE fit indices from AE moderation analyses for ADHD.

| **Sample** | **-2lnL** | | | | ***χ²* (*df*)** | | **AIC** | | | **BIC** | **SABIC** | |
| --- | --- | --- | --- | --- | --- | --- | --- | --- | --- | --- | --- | --- |
| MTP Hyperactivity/Inattention | |  | |  | |  | |  | | | |  |
| **Linear AE moderation**  Linear A moderation  No moderation | | | **17173.32**  17184.64  17190.52 |  | | **-**  11.32† (1)  17.20† (2) | | **17185.31**  17194.65  17198.52 | **17221.65**  17224.93  17222.74 | | | **17202.59**  17209.04  17210.03 |
| TBED-C Attention Problems | | | | | | | | | | | | |
| Linear AE moderation  **Linear A moderation**  No moderation | 5307.98  **5308.76**  5313.14 | | | | -  **.78 (1)**  5.16 (2) | | 5319.97  **5318.75**  5321.14 | | | 5349.25  **5343.15**  5340.66 | 5330.19  **5327.27**  5327.95 | |

†Significant change in chi-square at *p*<.05.

**Table S7:** Unstandardized path and moderation parameter estimates from AE models of ADHD.

| **Sample** | **Paths** | | | **Linear Moderators** |
| --- | --- | --- | --- | --- |
|  | **a e** | | | **A1 E1** |
| MTP Hyperactivity/Inattention  **Linear AE moderation**  Linear A moderation  No moderation  TBED-C Attention Problems  Linear AE moderation  **Linear A moderation**  No moderation | **.55***  **.63***  **.68***    **.70***  **.68***  **.75*** |  | **.80***  **.73***  **.73***  **.63***  **.66***  **.66*** | **.32* -.20***  **.13*** -  - -    .13 .07  **.18*** -  - - |

Bold font and asterisk, *p*<.05.

**Table S8:** Fit indices from nuclear twin family models.

| **Phenotype** |  | **AIC** | **BIC** | **SABIC** | **RMSEA** |  |
| --- | --- | --- | --- | --- | --- | --- |
| Anxious/Depressed | |  | | | |  |
| **ASFE** |  | **10736.09** | **10760.78** | **10744.89** | **.068** |  |
| ADFE |  | 10759.73 | 10784.41 | 10768.53 | .081 |  |
| ADSE 10741.49 10771.11 10752.06 .072 | | | | | |  |
| ASE |  | 10739.49 | 10764.18 | 10748.30 | .070 |  |
| AFE  ADE  AE |  | 10841.28  10750.09  10749.99 | 10861.03  10774.78  10769.74 | 10848.33  10758.90  10757.03 | .114  .076  .075 |  |
| Withdrawn/Depressed  ASFE 10802.50 10827.19 10811.31 .068 | | | | | |  |
| ADFE |  | 10774.23 | 10798.92 | 10783.04 | .047 |  |
| ADSE |  | 10775.74 | 10805.37 | 10786.31 | .048 |  |
| ASE 10776.72 10801.40 10785.52 .049 | | | | | |  |
| AFE  **ADE**  AE |  | 10868.51  **10773.74**  10777.37 | 10888.25  **10798.43**  10797.12 | 10875.55  **10782.55**  10784.41 | .099  **.046**  .049 |  |
| Somatic Complaints  ASFE |  | 10647.73 | 10672.42 | 10656.54 | .062 |  |
| ADFE |  | 10649.94 | 10674.62 | 10658.74 | .064 |  |
| ADSE |  | 10634.53 | 10664.16 | 10645.10 | .053 |  |
| **ASE 10632.53 10657.22 10641.34 .051** | | | | | |  |
| AFE  ADE  AE |  | 10801.84  10642.79  10650.38 | 10821.59  10667.47  10670.13 | 10808.88  10651.59  10657.43 | .127  .059  .063 |  |
| Attention Problems  ASFE  ADFE  ADSE  ASE  AFE  **ADE**  AE |  | 10783.79  10734.65  10734.37  10746.48  10906.44  **10732.52**  10769.93 | 10808.48  10759.34  10763.99  10771.17  10926.19  **10757.21**  10789.68 | 10792.60  10743.46  10744.93  10755.29  10913.49  **10741.33**  10776.98 | .078  .043  .043  .054  .126  **.041**  .069 |  |
| *Note*. The best-fitting model for a given set of analyses is highlighted in bold font, and is indicated by the lowest AIC, BIC, SABIC, and RMSEA values for at least 3 of the 4 fit indices. | | | | | |  |
|  |  |  |  |  |  |  |
|  |  |  |  |  |  |  |
|  |  |  |  |  |  |  |

**Table S9:** Unstandardized NTFM variance estimates from the best-fitting multigroup models at lower and higher disadvantage on the ADI.

| Outcome | ADI | Model | **A** | **D** | **S** | **F** | **E** | Assortative  mating | Passive rGE |
| --- | --- | --- | --- | --- | --- | --- | --- | --- | --- |
| Anxious/  Depressed | Lower | ASFE | .14  [.00,.45] | **-** | **.22***  [.04,.35] | .02  [.00,.10] | **.56***  [.45,.66] | **.14***  [.12,.15] | .05  [-.04,.07] |
|  | Higher |  | .10  [.00,.52] | **-** | **.22***  [.05,.32] | .07  [.00,.19] | **.60***  [.45,.68] | **.28***  [.25,.31] | **.08***  [-.03,.10] |
| Withdrawn/  Depressed | Lower | ADE | **.32***  [.23,.41] | **.17***  [.05,.32] | **-** | - | **.46***  [.35, .57] | **.11***  [.10, .13] | - |
|  | Higher |  | **.43***  [.33,.53] | .03  [.00,.21] | **-** | **-** | **.60***  [.45, .69] | **.10***  [.09, .11] | **-** |
| Somatic  Complaints | Lower | ASE | **.43***  [.33,.52] | - | **.14***  [.05,.22] | **-** | **.39***  [.31,.46] | **.11***  [.10,.13] | **-** |
|  | Higher |  | **.44***  [.33,.54] | - | .09  [.00,.19] | **-** | **.51***  [.41,.60] | **.16***  [.14,.18] | **-** |
| Attention  Problems | Lower | ADE | **.34***  [.24,.44] | **.20***  [.06,.32] | - | **-** | **.44***  [.34,.55] | **.09***  [.08,.11] | **-** |
|  | Higher |  | **.31***  [.21,.41] | **.32***  [.19,.45] | - | - | **.38***  [.30,.48] | **.16***  [.14,.17] | - |

*Note*. 95% confidence intervals are below the point estimates in brackets. A, D, S, F, and E represent the additive genetic influences, nonadditive genetic influences, environmental influences shared by siblings, family environmental influences, and nonshared environmental influences, respectively. Because A, D, S, F, and E are variances, neither their estimates nor their confidence intervals can be negatively signed. The passive rGE (gene-environment correlation) and assortative mating parameter estimates can be either positively or negatively signed. Bold font and an asterisk indicate that the parameter is significantly different than zero at *p*<.05.

**Table S10:** Biometric GxE fit indices for ADE models of callous-unemotional traits.

| **Phenotype** | **-2lnL** | ***χ²* (*df*)** | **AIC** | **BIC** | **SABIC** |
| --- | --- | --- | --- | --- | --- |
| Callous | | | | | |
| Linear ADE moderation | 3185.38 | - | 3201.38 | 3236.30 | 3210.90 |
| Linear E moderation  **No moderation** | 3185.62  **3189.34** | .24 (2)  **3.96 (3)** | 3197.62  **3199.35** | 3223.81  **3221.30** | 3204.76  **3205.30** |
| Uncaring | | | | | |
| Linear ADE moderation | 3190.80 | - | 3206.79 | 3241.71 | 3216.31 |
| **Linear E moderation**  No moderation | **3191.34**  3197.72 | **.54 (2)**  6.92 (3) | **3203.34**  3207.72 | **3229.53**  3229.54 | **3210.48**  3213.67 |
| Unemotional | | | | | |
| Linear ADE moderation | 3250.76 | - | 3266.76 | 3301.66 | 3276.26 |
| **No moderation** | **3251.30** | **.54 (3)** | **3261.31** | **3283.12** | **3267.25** |

†Significant change in chi-square at *p*<.05.

**Table S11:** Unstandardized path and moderation parameter estimates for ADE models of callous-unemotional traits.

| **Phenotype** | **Paths** | | | **Linear Moderators** |
| --- | --- | --- | --- | --- |
|  | **a d e** | | | **A1 D1 E1** |
| Callous  Linear ADE moderation  **No moderation**  Uncaring  Linear ADE moderation  **Linear E moderation**  No moderation  Unemotional  Linear ADE moderation  **No moderation** | **.56***  **.66***  .00  .00  .00  .00  .00 | .46~  .25  **.70***  **.75***  **.76***  **.56***  **.53*** | **.60***  **.71***  **.57***  **.54***  **.65***  **.80***  **.85*** | .12 -.24 .23  - - -  .00 .13 .19  - - .26~  - - -  .00 -.07 .10  - - - |

Bold font and asterisk, *p*<.05. ~, *p*<.10.


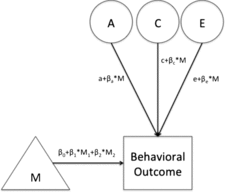


**Figure S1:** Path diagram of the full linear moderation model (Purcell, 2002). A, C, and E represent genetic, shared environmental, and nonshared environmental contributions, respectively, while M represents the moderator. For ease of presentation, the cotwin variables and paths are omitted here, although they are estimated in the model.

**References**

Achenbach, T. M., & Rescorla, L. A. (2003). Manual for ASEBA Adult Forms & Profiles.

Burt, S. A., Klump, K. L., Gorman-Smith, D., & Neiderhiser, J. M. (2016). Neighborhood disadvantage alters the origins of children’s nonaggressive conduct problems. *Clinical psychological science*, *4*(3), 511-526.

Grigorenko, E. L., Geiser, C., Slobodskaya, H. R., & Francis, D. J. (2010). Cross-informant symptoms from CBCL, TRF, and YSR: trait and method variance in a normative sample of Russian youths. *Psychological assessment*, *22*(4), 893.

Hayward, C., & Sanborn, K. (2002). Puberty and the emergence of gender differences in psychopathology. *Journal of Adolescent Health*, *30*(4), 49-58.

Hooper, D., Coughlan, J., & Mullen, M. (2008). Structural equation modelling: Guidelines for determining model fit. Articles, 2.

Keller, M. C., Medland, S. E., & Duncan, L. E. (2010). Are extended twin family designs worth the trouble? A comparison of the bias, precision, and accuracy of parameters estimated in four twin family models. *Behavior genetics*, *40*(3), 377-393.

Kind, A. J., & Buckingham, W. R. (2018). Making neighborhood-disadvantage metrics accessible—the neighborhood atlas. *The New England journal of medicine*, *378*(26), 2456.

Purcell, S. (2002). Variance components models for gene–environment interaction in twin analysis. *Twin Research and Human Genetics*, *5*(6), 554-571.

Rice, F., Harold, G. T., & Thapar, A. (2002). Assessing the effects of age, sex and shared environment on the genetic aetiology of depression in childhood and adolescence. *Journal of child Psychology and Psychiatry*, *43*(8), 1039-1051.

Singh, G. K. (2003). Area deprivation and widening inequalities in US mortality, 1969–1998. *American journal of public health*, *93*(7), 1137-1143.
